# Improving the Performance of Dye-Sensitized Solar Cells

**DOI:** 10.3389/fchem.2019.00077

**Published:** 2019-02-14

**Authors:** Gerrit Boschloo

**Affiliations:** Department of Chemistry-Ångström Laboratory, Uppsala University, Uppsala, Sweden

**Keywords:** mesoporous TiO_2_, recombination, electron lifetime, cobalt-complex, maximum power point, organic dyes

## Abstract

Dye-sensitized solar cells have been investigated intensively during the last three decades. Nevertheless, there are still many aspects to be explored to further improve their performance. Dye molecules can be modified endlessly for better performance. For instance, steric groups can be introduced to slow down recombination reactions and avoid unfavorable aggregation. There is a need for more optimal dye packing on the mesoporous TiO_2_ surface to increase light absorption and promote a better blocking effect. Novel redox mediators and HTMs are key elements to reach higher performing DSC as they can offer much higher output voltage than the traditional triiodide/iodide redox couple.

## Introduction

Dye-sensitized solar cells (DSCs) have attracted much attention in recent years, because of their good photovoltaic performance, specifically under low-light conditions, as well as their flexibility in terms of colors and appearance, their relatively simple fabrication procedures and their potential low cost. Efficient dye-sensitized solar cells (DSCs) were first developed in the 1990s marked by the breakthrough work by O'Regan and Grätzel (1991) who first used mesoporous TiO_2_ electrodes prepared from colloidal TiO_2_ nanoparticles (O'Regan and Grätzel, 1991).

**Figure 1:**
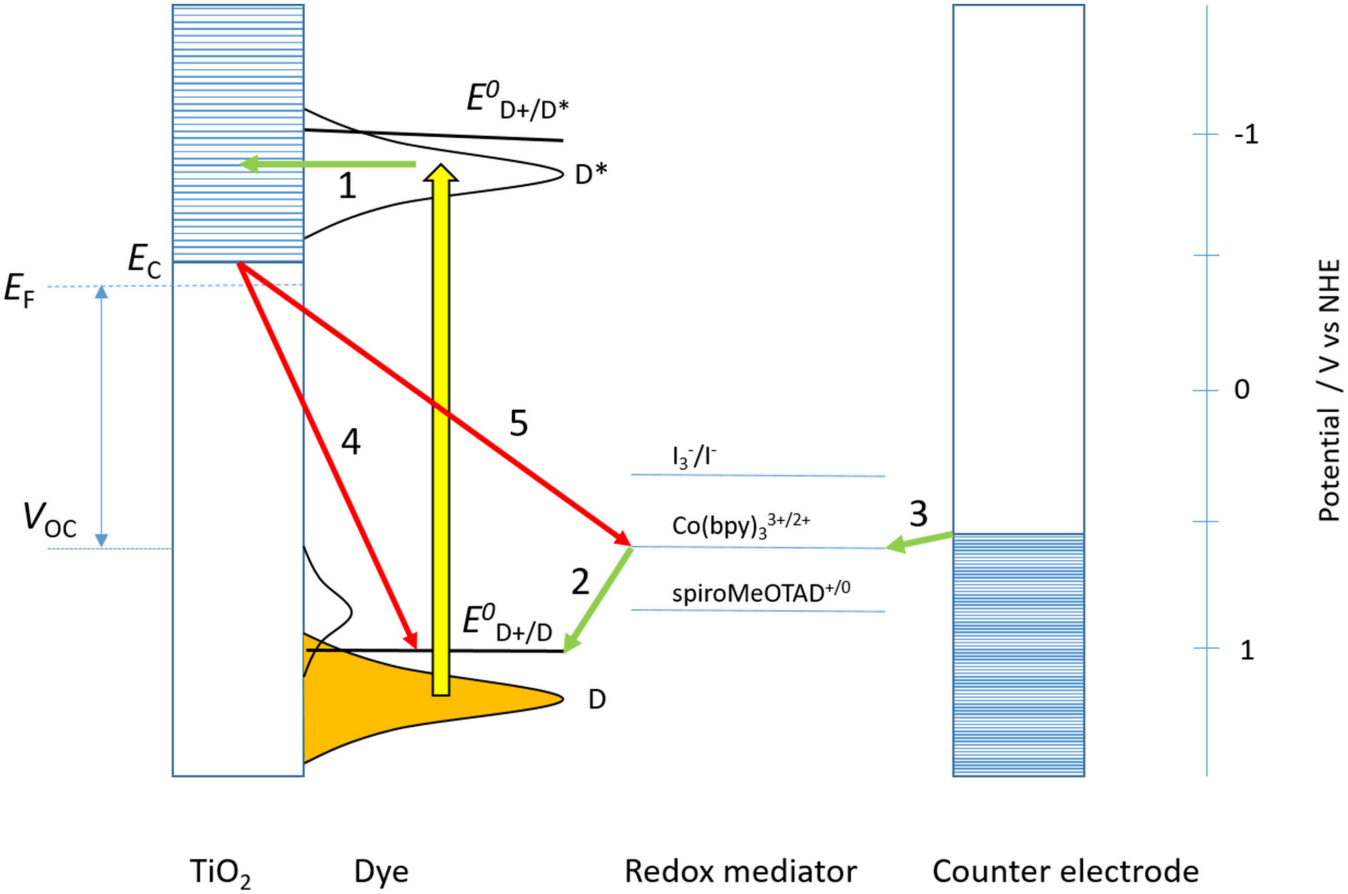
Energy scheme and working mechanism of a dye-sensitized solar cell. Electron transfer reactions are indicated with numbers and arrows (green for forward, red for recombination) that are referred to in the text. The energy levels of different redox mediators are indicated (spiro-MeOTAD is a solid-state hole conductor).

The working mechanism of DSC differs much from other types of solar cells (O'Regan and Grätzel, 1991; Hagfeldt et al., 2010). In their original conception, the DSC is a photoelectrochemical solar cell, consisting of a dye-sensitized mesoporous TiO_2_ working electrode (WE), a redox electrolyte and a counter electrode (CE). Both the WE and CE can be (semi) transparent, which allows for illumination of the solar cell from either side. Dye molecules, equipped with suitable anchoring groups, are adsorbed as a monolayer onto the mesoporous TiO_2_ electrode. When the dyes absorb light, the excited molecules can inject electrons into the conduction band of TiO_2_ (electron transfer (ET) reaction 1 in Figure 1). A redox mediator in the electrolyte regenerates the resulting oxidized dye molecules (ET 2). The oxidized form of the mediator is responsible for transport of positive charge to the counter electrode by means of diffusion. Finally, electrons in the TiO_2_ are collected at the underlying fluorine-doped tinoxide (FTO)-coated glass substrate and move through an external circuit to the counter electrode, where they reduce the oxidized redox mediator (ET 3), thus completing the cycle. A schematic presentation of these processes is shown in Figure 1.

A number of recombination reactions in the DSC compete with the forward processes described above. Radiative and non-radiative de-excitation of the dye competes with electron injection from the excited dye into the conduction band of TiO_2_. Furthermore, electrons in the TiO_2_ can recombine with oxidized dye molecules (ET 4) or to the oxidized form of the redox mediator (ET 5). For optimized DSC systems the incident photon to current conversion efficiency (IPCE) is about 90%. Since there are some reflection and transmission losses, this implies that every absorbed photon gives an electron in the external circuit under the measurement conditions used, typically under short-circuit conditions. Under operating conditions, when the solar cell delivers maximum power output (in the maximum power point, MPP), the IPCE is significantly lower and there are both current and voltage losses. In order to fully optimize the DSC these losses must be minimized, as will be discussed in section Limiting Factors in the DSC.

The triiodide/iodide (${\text{I}}_{3}^{-}$/I^−^) redox couple is most frequently used in DSCs. However, this complex redox couple has some serious limitation, as pointed out in our previous work (Boschloo and Hagfeldt, 2009). Specifically, its formal reduction potential (*E*^0^') is relatively negative (+0.35 V vs. NHE) compared to the *E*^0^' (D^+^/D) of typical dyes (located at ca. 1 V vs. NHE), resulting in a large loss of more than 0.5 V. The origin lies in the multistep regeneration mechanism that involves formation of the ${\text{I}}_{2}^{-}$ radical as a reaction intermediate. Furthermore, triiodide is quite strongly colored and there are issues with long-term stability (particles). In short, triiodide/iodide is not the avenue to DSC with higher performance. Several promising alternative redox mediators will be discussed in section Components for more efficient DSCs.

A disadvantage compared to other solar cell technologies is the use of a liquid redox electrolyte in efficient DSCs. This makes encapsulation problematic and it makes the devices less compatible with other solid-state photovoltaic technologies. The liquid redox electrolyte can, however, be replaced by a solid- state hole conductor in DSCs to make fully solid-state DSC (Bach et al., 1998). Unfortunately, this usually comes at the cost of faster recombination and a lower overall performance.

In order to make significant impact in the field of photovoltaics, the performance of DSC needs to be further improved. Their record efficiencies under 1 sun illumination, shown in Table 1, are just over 10%, which is lower than that of most other competing photovoltaic technologies. For indoor applications, however, DSC holds the record in performance with 32% at 1,000 lux (Cao et al., 2018). One reason is for this that the absorption spectrum of the dye can ideally match the emission spectrum of an indoor light source.

**Table 1:** Redox mediators and dyes used in high performance dye-sensitized solar cells.

**Redox couple (R^**+**^/R)**	***E*^**0**^'/V vs. NHE**	**Dye (type)**	**Selected record solar cell efficiencies (*V*_**OC**_, *J*_**SC**_, *FF*)**	**Year, reference (comment)**
I_3_-/I-	+0.35	Black dye (Ru complex) + Y1	11.9% (0.744 V, 22.5 mA cm^−2^, 0.712)	2011 (Han et al., 2012)
Co(bpy)${}_{3}^{3+/2+}$	+0.56	YD2-o-C8 (porphyrin) + Y123 (DpA)	12.3% (0.935 V, 17.7 mA cm^−2^, 0.74)	2011 (Yella et al., 2011)
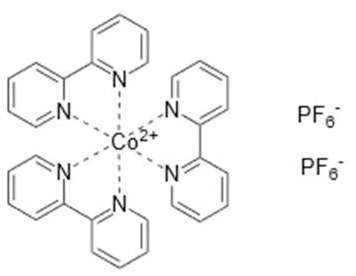		SM315 (porphyrin)	13.0% (0.91 V, 18.1 mA cm^−2^, 0.78)	2014 (Mathew et al., 2014)
		R6 (DpA)	12.6% (0.850 V, 19.7 mA cm^−2^, 0.754)	2018 (Ren et al., 2018)
Co(phen)${}_{3}^{3+/2+}$	+0.62	Adeka-1+ LEG4 (DpA)	14.3% (1.01 V, 18.3 mA cm^−2^, 0.771)	2015 (Kakiage et al., 2015) (open device)
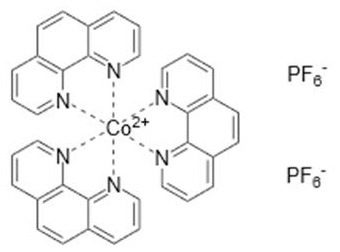				
Co(bpy-pz)${}_{2}^{3+/2+}$	+0.86	Y123	10.1% (0.998 V, 13.1 mAcm^−2^, 0.774)	2012 (Yum et al., 2012)
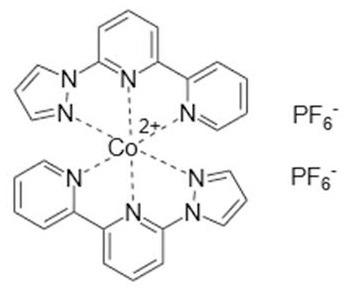				
Cu(tmp)${}_{2}^{2+/+}$ (TFSI)	+0.91	Y123	13.1% (1.05 V, 15.7 mA cm^−2^, 0.79)	2018 (Cao et al., 2018)
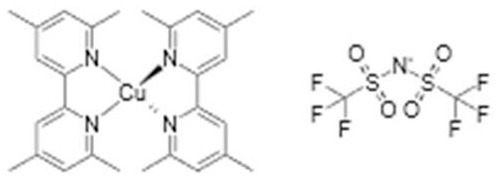				
Spiro-MeOTAD ^+/0^ HTM	+0.75	S5 (DpA)	7.81% (0.83 V, 12.9 mA cm^−2^, 0.73)	2017 (Shen et al., 2017)
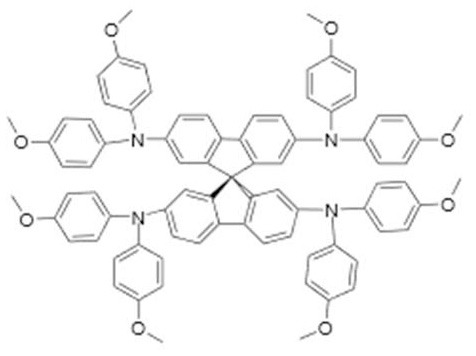				
Cu(tmp)${}_{2}^{2+/+}$ HTM	+0.91	WS72 (DpA)	13.8% (1.07 V, 11.7 mA cm^−2^, 0.79)	2018 (Zhang et al., 2018)

This perspective focusses most on the efficiency of DSCs, but in the end, their long-term stability is equally important for practical applications.

## Limiting Factors in the DSC

The Shockley-Queisser (SQ) limit gives the maximum efficiency attainable for a single junction photovoltaic device, which is 33.8% under 1,000 W m^−2^ solar irradiation with AM1.5G spectral distribution (Green, 2012). While this limit was derived for semiconductor devices, it is in principle also valid for DSCs. The bandgap *E*_g_ sets the range for light absorption: all photons with energies larger than *E*_g_ are absorbed and contribute to photocurrent, those with smaller energies are transmitted and not used. The only recombination processes that are considered in the derivation of the SQ limit are radiative processes, since these are unavoidable. Any non-radiative recombination processes will further lower the efficiency. According to the SQ analysis the optimal bandgap is 1.3 eV; the maximum obtainable efficiency gradually decreases to 25% for *E*_g_ = 1.9 eV. This would correspond to a dye with an absorption onset wavelength of 650 nm, a value commonly obtained for efficient sensitizing dyes in the DSC. The corresponding theoretical photocurrent under short-circuit conditions, *J*_SC_, would be 17 mA cm^−2^.

While the maximum open circuit potential (*V*_OC_) in semiconductor photovoltaics has *E*_g_/*e* as the absolute upper limit, for DSC the upper limit is set by the difference between the conduction band potential *E*_C_ of the TiO_2_ on one side and the redox potential of the electrolyte on the other side. This value will always be smaller than the “bandgap” of the dye. *E*_C_ of TiO_2_ anatase is about −0.5 V vs. NHE when its surface is uncharged (i.e., at neutral pH, without specific ion adsorption, and without electron accumulation). This level can be altered by additives in the electrolyte that lead to a change in the surface charge or alter the dipole moment at the semiconductor/electrolyte interface. Alternatively, *E*_C_ can be changed by chemical modification of the TiO_2_, for instance by incorporation of Mg in the structure, which can to shift *E*_C_ to more negative potential, to about −0.7 V vs. NHE (Kakiage et al., 2016). Ultra-thin metal oxide layers (such as Al_2_O_3_) covering the mesoporous TiO_2_ can also affect the location of *E*_C_ (Kay and Grätzel, 2002). Such layers also affect the kinetics of interfacial electron transfer reactions, and may be used for fine-tuning of the properties of the DSC.

The electrochemical potential of electrons in the TiO_2_, usually referred to as the Fermilevel *E*_F_, is given by:

$$\begin{array}{l} E_{F}=E_{C}-\frac{k_{B}T}{e}ln\frac{n_{c}}{N_{c}}\left(V\right) \end{array}$$

where, *k*_*B*_ is the Boltzmann constant, *T* the absolute temperature, *e* the elementary charge (*k*_*B*_*T*/*e* is 0.0257 V at room temperature), *n*_*c*_ is the density of conduction band electrons, and *N*_*c*_ is the effective density of electronic states at the bottom of the conduction band. *N*_*c*_ is a material constant and is about 10^20^ cm^−3^ for TiO_2_ anatase. Under illumination at open-circuit conditions *E*_F_ depends on *n*_c_, which depends in turn on the generation flux of injected electrons and the rate constants for electron recombination. As a rule of thumb, *E*_F_ is about 0.1 V more positive than *E*_C_ at 1 sun.

Formal reduction potentials of a series of redox mediators for DSC are listed in Table 1 along with obtained record efficiencies in DSCs. Initial work on DSCs focused on the ${\text{I}}_{3}^{-}$/I^−^ redox couple, which has very favorable electron transfer kinetics, giving very low recombination losses and high *J*_SC_. This comes, however, at the cost of a relatively low *V*_OC_ due to the rather negative value of the redox potential. Successful alternative redox mediators have more positive redox potential and can provide higher *V*_OC_. Nearly all of these redox mediators give, however, faster electron recombination to oxidized redox mediators, thereby lowering the Fermi level on the TiO_2_ under operational conditions.

The highest reported *V*_OC_ for a DSC is 1.4 V, and was obtained for a Mg-doped TiO_2_ with additional surface modification by MgO and Al_2_O_3_, sensitized by a coumarin dye, and in combination with the Br${}_{3}^{-}$/Br^−^ redox couple (Kakiage et al., 2016). The doping and surface modification of the TiO_2_ raised the conduction band edge to about −0.7 V vs. NHE, while the formal potential of the redox couple is about +0.9 V vs. NHE.

The best performing DSCs with Co(bpy)_3_ redox electrolyte can obtain a *V*_OC_ of about 0.9 V. The *V*_OC_ for best ssDSC with spiro-MeOTAD hole conductor is about 0.8 V. As the redox potentials of the Co and the spiro:MeOTAD hole conductor differs by about 0.2 V, this implies that Fermi level in TiO_2_ is about 0.3 eV lower in the mesoporous TiO_2_ for the ssDSC device under open circuit illumination conditions. This is due to much faster electron recombination kinetics. Using Equation 1, it can be estimated that the concentration of conduction band electrons is many orders of magnitude lower in the ssDSC than in the Co(bpy)_3_-DSC.

As indicated in Figure 1, the energy level of the ground state dye displays a Gaussian distribution, with an average energy that is below the formal redox energy by an amount equal to the reorganization energy λ. Upon excitation energy levels D^*^ should overlap with acceptor levels in the conduction band of TiO_2_ for efficient electron injection. A lower value of λ would allow for closer matching between *E*_C_ of the semiconductor and the calculated standard potential for the excited dye, *E*^0^(D^+^/D^*^) and less voltage loss in the DSC. The reorganization energy of the dye is due to internal molecular reorganization of the dye when it changes redox state and external reorganization of the solvent shell. Due to the absence of solvent, reorganization energies should be lower in solid-state DSC.

The electron injection (reaction 1 in Figure 1) competes with radiative and non-radiative decay processes of the excited dye. A long-lived excited state of the dye it therefore favorable. Excessive energy losses during the injection process should, however, be avoided (Haque et al., 2005). After injection the electrons travel through the mesoporous film and are collected at the FTO substrate. Electron recombination to oxidized dye (reaction 4) and the oxidized form of the redox mediator (reaction 5) must be avoided. The electron lifetime is the inverse of the sum of the rate constants of both recombination processes. It is frequently assumed that electron recombination to the oxidized dye in negligible, as the dye regeneration is usually rather fast on a microsecond time scale (reaction 3). Under solar cell operation conditions, however, a large concentration of electrons in accumulated in the mesoporous TiO_2_, which will accelerate the recombination process significantly (Haque et al., 2000). Haque et al. determined that the halftime for recombination to oxidize Ru-dye (N3) decreased to about 1 ns when a potential of −0.3 V vs. NHE was applied on the mesoporous TiO_2_ electrode (Haque et al., 2000). Therefore, significant recombination to the oxidized sensitizer can take place in the dye-sensitized solar operating at MPP conditions.

The kinetics of electron recombination to the redox electrolyte depends very strongly on the nature of the oxidized form of the redox mediator. It is very slow for triiodide, faster for cobalt mediators and faster still for triphenylamine based mediators or hole conductors. Crucial for the successful use of the latter two is that kinetics can be slowed down by structural modification of the dye: group can slow down the recombination process.

**Figure 2:**
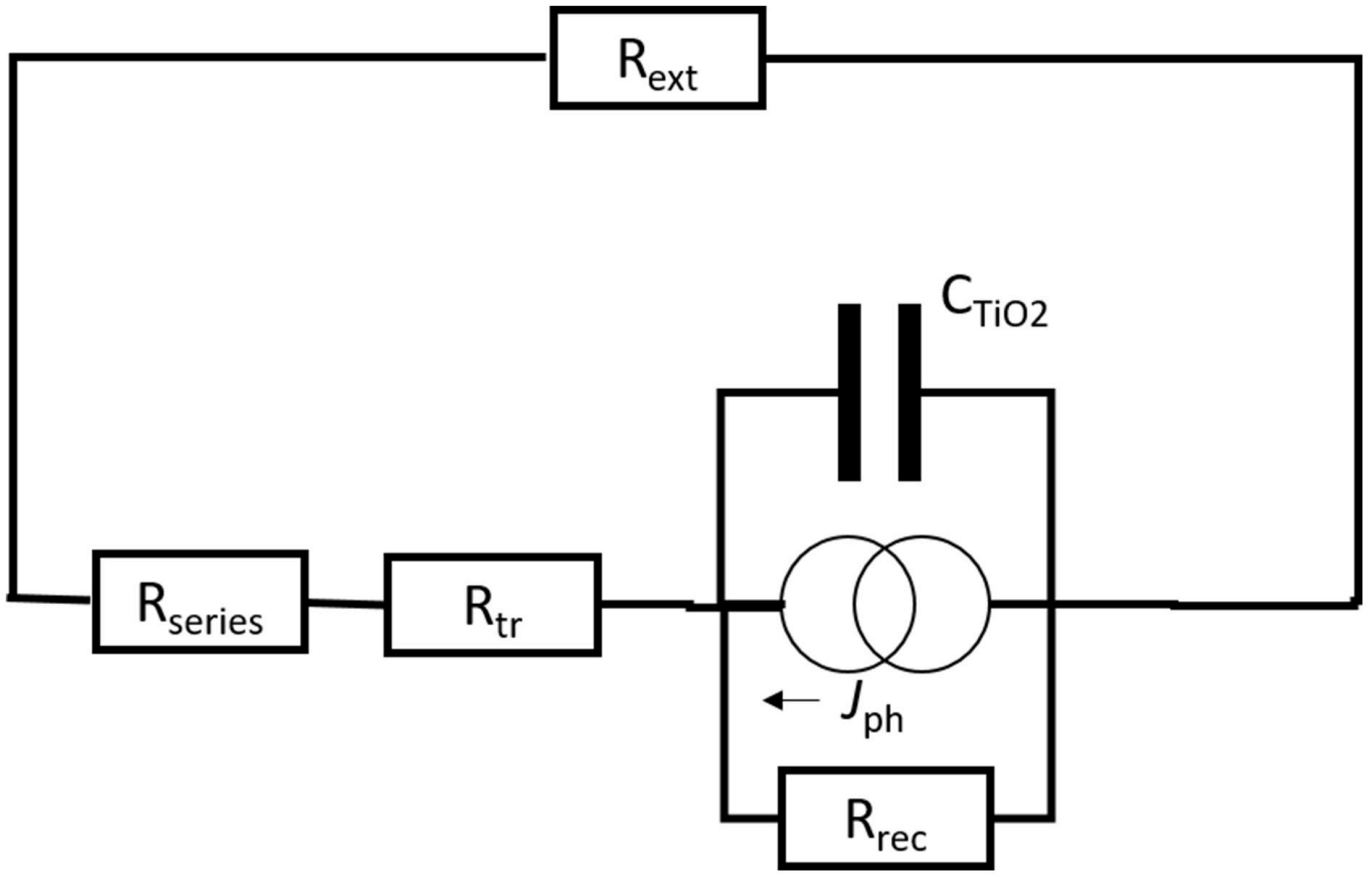
Schematic electrical diagram of a dye-sensitized solar cell. A current generator give a photocurrent *J*_ph_, which charges the capacitor. The current flow is through *R*_series_ + *R*_tr_ + *R*_ext_ and through *R*_rec_.

A long electron lifetime is favorable for the DSC as it will improve the *V*_OC_. In regular liquid-electrolyte DSCs the electron lifetime is typically 1–10 ms under open-circuit conditions and one sun illumination. The electron transport time should be smaller than the lifetime to avoid losses during transport. Typical values under short-circuit conditions are about one order of magnitude smaller than the electron lifetime. Under operational condition (at MPP) the electron transport in the mesoporous TiO_2_ is not a limiting factor, since transport becomes faster upon more electron accumulation, resulting in a more negative potential for *E*_F_. In other words, the transport resistance *R*_tr_ becomes smaller when the TiO_2_/electrolyte capacitor *C*_TiO2_ is charged, see Figure 2.

Under open-circuit conditions the external resistor *R*_ext_ is infinite. All current then goes through the recombination resistance *R*_rec_. This is the normal condition to measure electron lifetime τ, which is equal to *R*_rec_ × *C*_TiO2_. Under short circuit conditions *R*_ext_ is equal to 0. The charge collection efficiency : η_CC_ is then given by *R*_rec_/(*R*_series_ + *R*_tr_ + *R*_rec_), where *R*_series_ is the series resistance discussed below. Under MPP-conditions we find: η_CC_ = *R*_rec_ /(*R*_series_ + *R*_tr_ + *R*_ext_ + *R*_rec_).

Based on this simple scheme, it is evident that the recombination resistance must be maximized to increase current collection efficiency and output voltage across the external resistor under MPP conditions. The recombination resistance will decrease with increasing thickness and the surface area of the mesoporous electrode. Increasing thickness will, however, increase the generated photocurrent. For every specific DSC system there is an optimum film thickness: Typical values are ~10 μm for regular liquid electrolyte DSC and ~2 μm for solid-state DSCs.

Some series resistance losses are unavoidable in practical DSC devices. There will be some resistance due to the FTO substrates that are used, due to the charge transfer resistance at the counter electrode and due to diffusion resistance in the electrolyte. Han et al. make a detailed analysis of resistances in the DSC, and minimized *R*_series_ down to 1.8 Ohm cm^−2^ by optimizing the catalytic performance of the counter electrode and the electrolyte distance between working and counter electrode (Han et al., 2005). By minimizing the distance between WE and CE the diffusion resistance in the electrolyte is minimized. There is, however, still a remaining resistance due diffusion of the redox mediator in the pores of the working electrode. Short-circuiting between WE and CE must also be avoided. The use of PEDOT on the counter electrode seems to prevent short circuiting (Cao et al., 2018).

## Components for More Efficient DSCs

### Mesoporous Metal Oxide Electrodes

Mesoporous TiO_2_ (anatase) is by far the most used wide-bandgap semiconductor electrode used in DSC, and so far the most successful. In several studies the nanoparticle size, film porosity, and TiCl_4_ after treatment (Ito et al., 2008) have been optimized, but the optimal parameters for a specific dye-sensitized solar cell system depends strongly on dye and redox mediator system. For instance, more porous mesoporous films are best for DSCs with cobalt complex as redox mediator. An additional reflective TiO_2_ layer is usually added on top of a transparent TiO_2_ layer for improved light harvesting (Ito et al., 2008). Alternatively, reflecting particles (Wang et al., 2004) or voids (Hore et al., 2005) can be incorporated into the mesoporous film. Surface modification with ultra-thin metal oxides can be beneficial (Kay and Grätzel, 2002; Kakiage et al., 2015). Nevertheless, some properties of TiO_2_ are not ideal: TiO_2_ is a well-known photocatalyst. The bandgap of TiO_2_ anatase is 3.2 eV, which implies that light below 390 nm can excite the semiconductor, which leads to highly reactive holes. The holes may lead the destructive oxidation reactions with organic components of the DSC. UV filters are therefore generally used for long-term stability studies of DSC devices under full sunlight conditions. There are ways to minimize the photocatalytic action of TiO_2_, for instance by addition of an ultrathin layer of Al_2_O_3_ or MgO (Kay and Grätzel, 2002).

The use of a mesoporous semiconductor with a higher bandgap is advantageous. SnO_2_ with *E*_g_ of 3.6 eV will not absorb as much UV light from the solar spectrum. SnO_2_ has successfully used been used in DSC, but its *E*_C_ is located at more positive potential by about 0.5 V compared to TiO_2_, which limits its performance due to low voltage output. By covering the SnO_2_ with an ultrathin metal oxide layer (such as ZnO, Al_2_O_3_, or MgO) a much improved voltage can be obtained (Kumara et al., 2001; Kay and Grätzel, 2002).

ZnO has been studied intensively as a nanostructured electrode in a wide variety of morphologies in the DSC (Zhang et al., 2009). Although there are claims that its better electron transport properties should make ZnO a better nanostructured electrode for DSC, no improved performance is found compared to the traditional mesoporous TiO_2_ electrodes. This is because the electron transport is not a limiting factor for the DSC under operational conditions (at the MPP). In general, other metal oxides can function a mesoporous electrode in DSC devices, but so far their performance is lower than that of their TiO_2_ counterparts.

### Dyes

**Figure 3:**
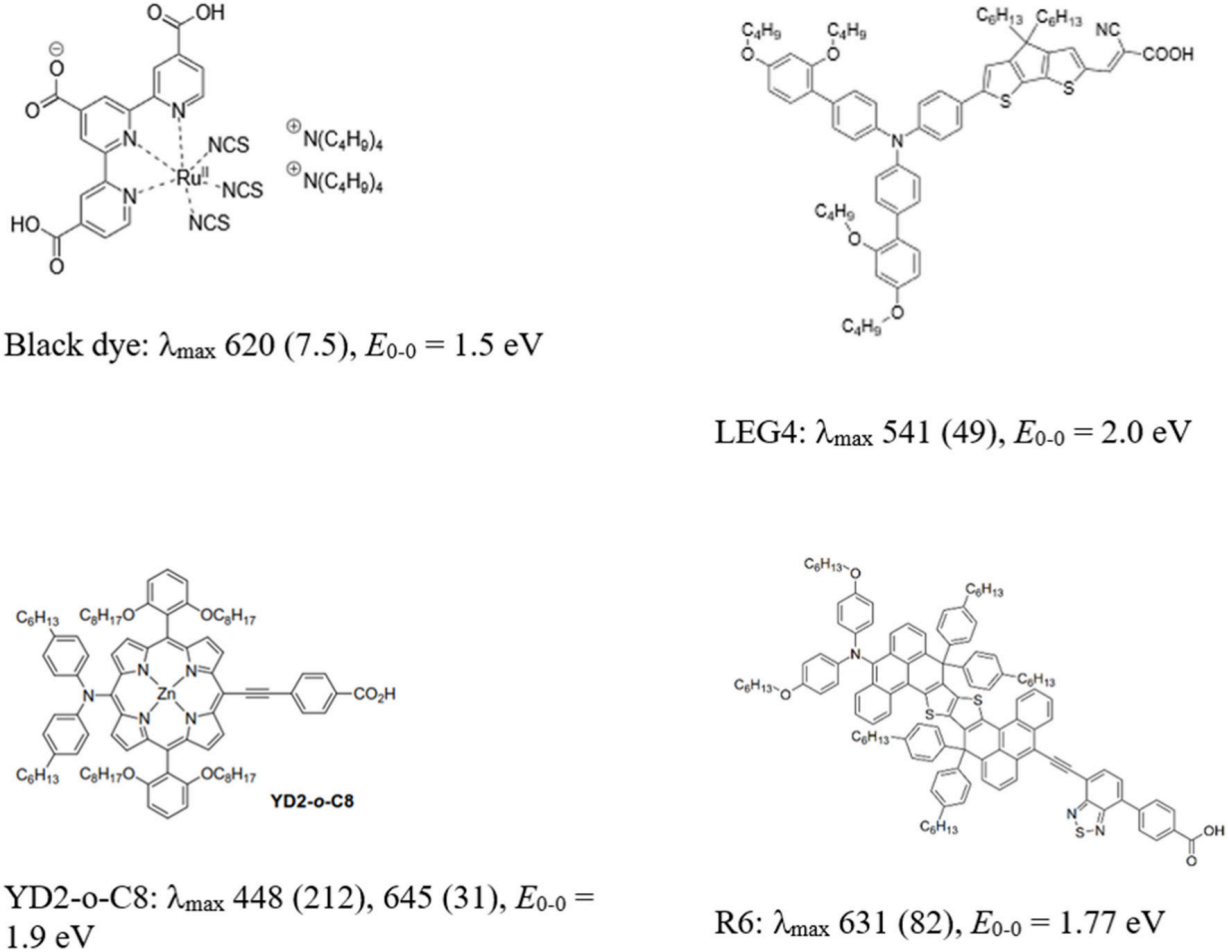
Structures of efficient molecular sensitizers for DSC, with absorption maximum (λ_max_) in nm, extinction coefficient (in 10^3^ M^−1^ cm^−1^) and zero-zero transition energy (E_0−0_). LEG4 is structurally nearly identical to Y123, having butoxy-groups instead of hexoxy.

As will be discussed later, there is a requirement of thinner mesoporous TiO_2_ electrodes set by new redox mediators for DSC and hole conductors for ssDSC. Therefore, there is a need for dyes with higher extinction coefficients than the traditional Ru-complex based dyes. Organic dyes are therefore preferred, as is reflected in Table 1 with best performing DSCs. Structures and some absorption parameters of selected dyes are displayed in Figure 3. Many successful organic dyes have a donor-pi-acceptor (DpA) structure, which leads to electron density movement toward the acceptor part upon photoexcitation. Typically, the binding group is incorporated in the acceptor part, as in the case of cyanoacrylic acid.

The dye structure has an important role in their overall performance. A good blocking behavior is crucial for successful use in combination with the novel redox mediators or hole conductors. Steric groups can slow down the electron transfer between TiO_2_ and oxidized redox mediator or hole conductor (Feldt et al., 2010).

A further requirement for optimized performance is that of dyes with relatively long excited state lifetime and high fluorescence yield. Any fast deactivation pathways should be avoided as it will affect the performance negatively. If the excitation lifetime is longer, the injection efficiency will increase. Wang et al. developed a series of highly fluorescent organic dyes with improved lifetimes, such as the R6 with an fluorescent lifetime of 260 ps for R6 adsorbed on mesoporous Al_2_O_3_ film (Ren et al., 2018).

Co-sensitization is a successful way to improve performance of the DSC. Record devices are usually based on co-sensitized solar cells, see Table 1. Strong and panchromatic light absorption can be achieved by selecting suitable dyes. In several cases, co-sensitization has the beneficial effect of decreasing dye aggregation (Ogura et al., 2009; Hao et al., 2016a). Furthermore, a higher dye load can be obtained. An interesting approach is to use dyes with different binding groups that do not compete for the same binding sites on TiO_2_. Shibayama et al. successfully combined the black dye (with carboxylic acid binding groups) with an organic dye possessing a pyridine binding group (Shibayama et al., 2014). They demonstrated that the dyes adsorbed to different binding sites.

### Redox Mediators

Cobalt-based redox mediators have been tested for DSC since 2001 (Nusbaumer et al., 2001; Sapp et al., 2002) but their breakthrough came later in 2010 by the work of Feldt et al. (2010) who first demonstrated efficient DSCs with cobalt complex based redox mediators by selecting dyes with suitable properties. In contrast to the triiodide/iodide system, cobalt complexes can exhibit a wide range of redox potentials, depending on the chemical structure of their ligands. Variation of counter ions of these complexes is also important: this can strongly affect solubility in different solvents. It allows, for instance, for use of these redox mediators in water-based electrolytes (Ellis et al., 2016). Interestingly, good stability has been reported for electrolytes based on water (Ellis et al., 2016), or containing large concentrations of water (Law et al., 2010), which may open up for more environmentally friendly solar cell devices. The structures and redox potentials of cobalt complexes are shown in Table 1.

**Figure 4:**
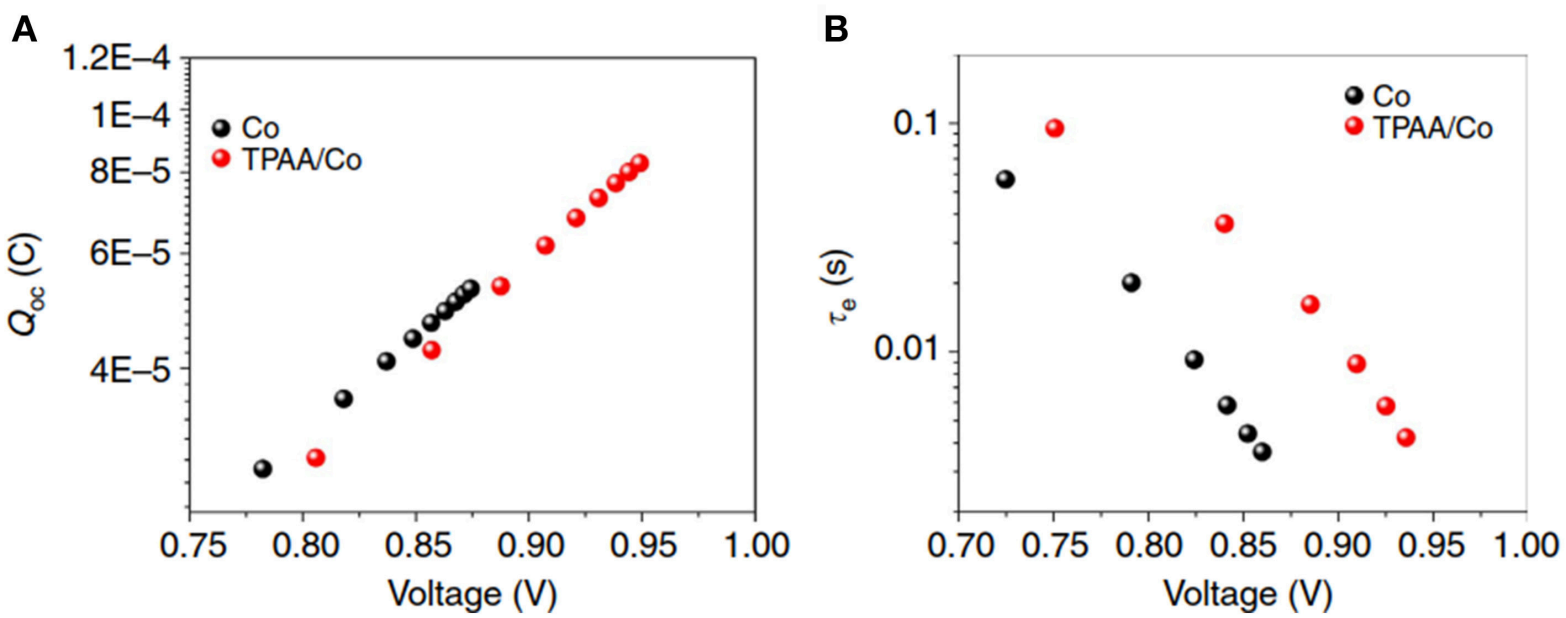
Effect of addition of a TPA electron donor to Co(bpy)_3_ electrolyte in a DSC. **(A)** Charge extraction and **(B)** electron lifetime measurements as function of *V*_OC_ in a DSC with and without additive (Hao et al., 2016b).

Recent investigations in our group clearly point to a problem of the present generation of cobalt redox mediators: they have relatively slow electron transfer kinetics (Hao et al., 2016b). The slow reduction of Co^3+^ species is favorable, as it gives slow kinetics for electron recombination with Co^3+^. On the other hand, the relatively slow regeneration of oxidized dye molecules by Co^2+^ is a critical issue that will limit the DSC performance. This was not recognized in earlier work of cobalt-based DSC. Addition of a rapid electron donor, such as a triphenylamine TPA, leads to very rapid regeneration of the oxidized dye molecules, which is on the sub-ns timescale (Hao et al., 2016b). The oxidized donor is in turn reduced by the Co^2+^ species. As a result, much higher *V*_OC_ was found and a marked increase in the electron lifetime, see Figure 4. The recombination between electron in TiO_2_ and oxidized dye molecules is strongly suppressed by the TPA additive, demonstrating that there was much recombination without this additive. As a result, the *V*_OC_ increased by about 100 mV, while the charge extraction experiment showed that the band edge level of the TiO_2_ was unchanged.

Interestingly, addition of the electron donor leads to an intermediate redox level in the DSC. This is not unlike the situation of the triiodide/iodide electrolyte, where the diiodide radical gives an intermediate redox level (Boschloo and Hagfeldt, 2009).

Another recent redox mediator development, largely driven by our research group, is that of the use of copper complexes (Freitag et al., 2015, 2016; Saygili et al., 2016). These mediators display faster dye regeneration kinetics compared to cobalt complexes (Freitag et al., 2016). Unexpectedly, relatively long electron lifetimes were observed. Very recent research from Hamann demonstrated that the Cu^2+^ state is chemically unstable and a Cu complex with 4-tert butylpyridine is formed, having a more positive potential and displaying slow electron transfer kinetics (Wang and Hamann, 2018).

## Solid-State DSCs

In order to make solid-state DSCs, the liquid redox electrolyte can be replaced by a solid hole transporting material (HTM). Most promising results have been achieved with molecular organic hole conductors such as spiro-MeOTAD, conduction polymers such as PEDOT, and very recently, with metal complexes (Freitag et al., 2015).

Organic small-molecular HTMs like spiro-MeOTAD give very rapid dye regeneration in the picosecond regime. While pore filling can be an issue, usually full contact between dye and HTM can be achieved for relatively thin mesoporous TiO_2_ films, even when the pore filling fraction is well below 100% (Snaith et al., 2008; Cappel et al., 2009; Melas-Kyriazi et al., 2011). The main limitations of ssDSC arises from the very fast recombination between electrons in TiO_2_ with holes in the HTM (Snaith et al., 2008; Melas-Kyriazi et al., 2011). This limits the performance of ssDSC by lowering the output voltage significantly.

The surprising finding that dried-out DSC with Cu-complex redox electrolyte were still functioning as efficient solar cells, led to new type of ssDSC, the so-called zombie solar cell (Freitag et al., 2015). Recent optimization led to a record efficiency of 11.7% so far (Zhang et al., 2018). Unlike other HTMs, relatively long electron lifetimes are found and relatively thick TiO_2_ films can be used. The precise nature of the amorphous dried-electrolyte HTM, containing Cu(tmbpy)_2_, LiTFSI, and perhaps 4-tert butylpyridine needs to be explored.

In general, ssDSC are highly attractive for practical application, but their performance needs to be improved. The introduction of an intermediate redox level may be helpful: it could remove the formed holes rapidly away from the TiO_2_/dye interface.

## Concluding Remarks

Despite three decades of intense research on dye-sensitized solar cells, there are still many aspects to be explored to further improve their performance. Nearly infinite types of modifications of dye molecules are possible, where steric groups can be introduced to slow down recombination reactions. There is a need for more optimal dye packing on the TiO_2_ surface to increase light absorption and to achieve a better blocking effect. Co-sensitization offers good possibilities in this respect. Novel redox mediators and HTMs are key to higher performing DSC as they can offer much higher output voltage than the traditional triiodide/iodide redox couple. High-performance DSC are of interest for many applications, ranging from power source for consumer electronics, to building integrated PV and large-scale power generation. The option of high transparency in the near-infrared region also opens up for the use of DSC as a top cell in tandem solar cells devices.

## Author Contributions

The author confirms being the sole contributor of this work and has approved it for publication.

### Conflict of Interest Statement

The author declares that the research was conducted in the absence of any commercial or financial relationships that could be construed as a potential conflict of interest.
